# Modifications in Organic Acid Profiles During Fruit Development and Ripening: Correlation or Causation?

**DOI:** 10.3389/fpls.2018.01689

**Published:** 2018-11-20

**Authors:** Willian Batista-Silva, Vitor L. Nascimento, David B. Medeiros, Adriano Nunes-Nesi, Dimas M. Ribeiro, Agustín Zsögön, Wagner L. Araújo

**Affiliations:** ^1^Departamento de Biologia Vegetal, Universidade Federal de Viçosa, Viçosa, Brazil; ^2^Max-Planck Partner Group at the Departamento de Biologia Vegetal, Universidade Federal de Viçosa, Viçosa, Brazil

**Keywords:** carbon metabolism, development, fruit, metabolism, organic acids, primary metabolism, ripening

## Abstract

The pivotal role of phytohormones during fruit development and ripening is considered established knowledge in plant biology. Perhaps less well-known is the growing body of evidence suggesting that organic acids play a key function in plant development and, in particular, in fruit development, maturation and ripening. Here, we critically review the connection between organic acids and the development of both climacteric and non-climacteric fruits. By analyzing the metabolic content of different fruits during their ontogenetic trajectory, we noticed that the content of organic acids in the early stages of fruit development is directly related to the supply of substrates for respiratory processes. Although different organic acid species can be found during fruit development in general, it appears that citrate and malate play major roles in this process, as they accumulate on a broad range of climacteric and non-climacteric fruits. We further highlight the functional significance of changes in organic acid profile in fruits due to either the manipulation of fruit-specific genes or the use of fruit-specific promoters. Despite the complexity behind the fluctuation in organic acid content during fruit development and ripening, we extend our understanding on the importance of organic acids on fruit metabolism and the need to further boost future research. We suggest that engineering organic acid metabolism could improve both qualitative and quantitative traits of crop fruits.

## Introduction

True fruits are specialized plant organs found solely in angiosperms (i.e., flowering plants), and these unique organs are believed to have evolved to improve seed dispersal and protection (Karlova et al., 2014). The natural diversity of angiosperms, ranging from small herbs to massive trees, coupled with their extraordinary ability to grow in a wide variety of habitats, has resulted in their intimate association with humans. This is particularly true considering the human connection with the fruits and seeds of flowering plants and their economic and nutritional value. Although fruits are usually characterized as derived from a mature ovary containing seeds, many structures frequently called ‘fruit’ are, in fact, composed of a variety of other flower tissues types (Seymour et al., 1993, 2013).

The countless types of fruits present in angiosperms can be operationally organized within a few broad categories by using combinations of traits such as: (i) dehiscence or indehiscence; (ii) fleshy or dry exterior; and free (apocarpous) or fused (syncarpous) carpels (Seymour et al., 2013). These variations are further exemplified, for instance, by fleshy fruits, which have evolved by an enlargement of seed-surrounding tissues to create attractive flesh for seed-dispersing animals. Dry fruits, on the other hand, have a dry mesocarp that normally needs to open in order to release the seeds inside via mainly abiotic dispersal mechanisms (Fuentes and Vivian-Smith, 2009). It is tempting to suggest that this high diversity in fruit types is adaptive and associated to specific dispersers. This fact apart, the existence of significant correlations between fruit type and habitat conditions in angiosperms indicates that the evolution of fruit fleshiness is more likely associated with changes in vegetation habitats than in dispersers itself (Bolmgren and Eriksson, 2005). Both explanations are not mutually exclusive. In any case, fleshy fruit evolution is an important and continually recurring theme in the study of flowering plant evolution. However, caution should be exercised when making assumptions with respect to the adaptive value of particular fruit traits (Niklas, 2016).

Developmental stages of fruits can be divided in: (i) fruit set; (ii) growth; (iii) maturation; and (iv) ripening. Fruit set occurs during and after fertilization, which can be defined as the transition of a quiescent ovary to a rapidly growing young fruit and depends on the successful completion of pollination and fertilization (Hamamura et al., 2012). Additionally, in the absence of pollination and successful fertilization, levels of hormones such as auxins and gibberellins drop and the flower begins a terminal phase of senescence, ending in floral abscission – an effect that was closely associated with cellular pH in the abscission zone cells (Sundaresan et al., 2014). Parthenocarpy, another physiological event occurring in the absence of pollination, is characterized by intensive alterations of phytohormones such as auxin, gibberellin (GA), cytokinin or combinations thereof during fruit set (McAtee et al., 2013). In fact, exogenous application of these phytohormones alone can trigger fruit development including fruit set and fruit growth, to a certain extent, and their combinations would induce a normal fruit growth in the absence of fertilization (Srivastava and Handa, 2005; Mignolli et al., 2018). Accordingly, increased GA content or perception are associated with parthenocarpic fruits in tomato (*Solanum lycopersicum* L.) mutants such as *pat* (Mazzucato et al., 1998), *pat-2* (Fos et al., 2003), *pat-3/4* (Fos et al., 2001), whereas facultative parthenocarpic fruits are observed in the *procera* mutant (Carrera et al., 2012). Not only gibberellin but also auxin has been determinant in parthenocarpy in tomato fruits as shown in the mutants *pin4* (Mounet et al., 2012) *arf7* (De Jong et al., 2009), *arf8* (Goetz et al., 2007), *iaa9* (*entire*) (Wang et al., 2005). On the other hand, when pollination and fertilization take place, a cascade of events is triggered, leading to development of seeds and fruit growth.

During fruit growth, a signal, most likely derived from seeds (sources and sinks for cytokinin and auxin), induces neighboring tissues to expand, by both cell division and expansion, with a positive correlation between seed number and fruit size (Bohner and Bangerth, 1988). This fact apart, polyploidy, which is associated with cell expansion, is another important feature involved in the determination of fruit weight and size in tomato (Cheniclet et al., 2005). Additionally, there is a concomitant accumulation of storage products and sugars (Carrari and Fernie, 2006). Fruit maturation begins when growth stops, reaching the competence to ripen, but the ripening process itself is a subsequent step. Ripening is a complex process whereby several metabolic changes related to softening and flavor characteristics as well as organoleptic traits take place (Lira et al., 2016). The precise transition between all the stages of fruit development, including maturation and ripening, requires a high amount of energy. This energetic demand is provided by metabolic adjustments on the abundance of different classes of carbon compounds (e.g., organic acids, amino acids, and sugars) during development (Osorio et al., 2013). These metabolic changes from normal development toward fruit ripening are coupled with a generally brief stage of accelerated ripening that is normally associated with enhanced respiration (Osorio et al., 2013; Cosme Silva et al., 2017).

Fleshy fruits are characterized by a broad range of sizes, shapes, and colors. Moreover, different species presents unique flavor characteristics that are of pivotal importance in several processes. Such aspects are attractive to frugivorous animals, enhancing seed dispersal, and furthermore have become an indispensable part of the human diet (Barry and Giovannoni, 2007; Karlova et al., 2014). Fleshy fruits are quite diverse, ranging from grapes (*Vitis vinifera* L.) and tomatoes, which are derived from the ovary, (the so-called true fruits), through apples (*Malus domestica* L. Borkh) and pineapples (*Ananas comosus* L. Merril), to strawberries (*Fragaria x ananassa* Duch.), which are derived from the receptacle tissues or from expansion of the sepals (called pseudo- or accessory fruits) (Barry and Giovannoni, 2007).

Fleshy fruits have traditionally been classified as climacteric or non-climacteric, based on physiological differences observed within their respiratory pattern and reliance on ethylene biosynthesis during ripening. Climacteric fruits, such as apple, banana (*Musa paradisiaca* L.), papaya (*Carica papaya* L), and tomato (further details in Table 1) show an increase in respiration and ethylene production at the onset of the ripening process (Cherian et al., 2014; Karlova et al., 2014). On the other hand, non-climacteric fruits, such as citrus (*Citrus* spp.), grapes, melon (*Cucumis melo* L.), and strawberries (*Fragaria* spp.) do not show the respiratory burst and ethylene production remains at a basal level during the whole fruit development including maturation and ripening (Giovannoni, 2004; Cherian et al., 2014). During maturation, fruits go through dramatic transformations in color, aroma, nutrient composition, flavor, and firmness. Additionally, during this process, the production of reactive oxygen species plays an important role, for instance, in the biosynthesis of carotenoids and in the transformations of chloroplasts to chromoplasts (Li and Yuan, 2013). Barsan et al. (2012) have shown an intriguing metabolic shift coupled with disrupted thylakoid biogenesis machinery and elevated energy production during tomato fruit ripening. These authors have also shown a strong decrease in the abundance of proteins of light reactions (photosynthesis, Calvin cycle, and photorespiration) and carbohydrate metabolism (starch synthesis/degradation), mostly between breaker (∼35 days after anthesis) and red stages (55 days after anthesis), as well as an increase in terpenoid biosynthesis (including carotenoids) and stress-response proteins (ascorbate-glutathione cycle, abiotic stress, redox, and heat shock). All these transformations are the result of complex and dynamic processes that involve a series of molecular and biochemical changes under genetic regulation and/or in response to environmental perturbations (Osorio et al., 2013).

**Table 1:** Main sugars and organic acid found in both climacteric and non-climacteric ripe fruits.

Fruits	Main sugar	Main organic acid	Reference
**Climacteric**			
Apple	Fructose	Malate	Wu et al., 2007; Zhang et al., 2010
Apricot	Glucose/Fructose	Malate/Citrate	Gurrieri et al., 2001; Fan et al., 2017
Atemoya	Fructose/Glucose	Fumarate/Malate	Alique and Oliveira, 1994; Anaya-Esparza et al., 2017
Banana	Fructose	Malate	Morvai and Molnár-Perl, 1992
Blueberry	Glucose/Fructose	Citrate	Ayaz et al., 2001; Perini et al., 2018
Guava	Fructose	Citrate	Bashir and Abu-Goukh, 2003
Mango	Fructose	Citrate/Malate	Medlicott and Thompson, 1985; Cosme Silva et al., 2017
Papaya	Glucose	Citrate	Selvaraj et al., 1982; Souza et al., 2014
Peach	Glucose/Fructose	Malate/Citrate	Morvai and Molnár-Perl, 1992; Cirilli et al., 2016
Pear	Fructose/Sorbitol	Malate/Citrate	Zhen et al., 2016
**Non-climacteric**			
Blackberry	Fructose	Isocitrate	Fan-Chiang and Wrolstad, 2010
Grape	Glucose	Malate	Martínez-Esteso et al., 2011
Lemon	Fructose	Citrate	Asencio et al., 2018
Lima	Fructose	Citrate	Albertini et al., 2006; Asencio et al., 2018
Lychee	Sucrose/Glucose	Tartaric/Malate	Harvey et al., 1975
Longan	Sucrose/Fructose	Malate/Oxalate	Yang et al., 2009
Orange	Fructose	Citrate	Albertini et al., 2006
Pineapple	Sucrose/Fructose	Citrate	Luengwilai et al., 2018
Ponkan	Sucrose/Fructose	Citrate/Quinate	Albertini et al., 2006; Lin et al., 2015
Strawberry	Fructose/Glucose	Citrate	Lee et al., 2018

Due to their economic importance, organoleptic traits are recurrent object of investigations seeking to improve fruit quality (Chen et al., 2012; Tieman et al., 2017). Among the several characteristics that are clearly important for fruit quality, such as nutritional and sensorial quality (e.g., visual aspect, firmness, and taste), palatability is assumedly of major metabolic significance, once this trait is mainly dependent on the balance between organic acids (acidity) and sugar (sweetness) levels (Kader, 2008; Brasil and Siddiqui, 2018). These two classes of metabolites are directly connected to central carbon metabolism, where they are also involved in the biosynthetic route of diverse compounds such as amino acids, vitamins, and terpenic aroma volatiles, which influence fruit aroma (Lin et al., 2015; Beauvoit et al., 2018). The biochemical changes underlying fruit ripening and its regulation have been extensively studied in different fruit types (Giovannoni, 2001, 2004; Barry and Giovannoni, 2007; Osorio et al., 2013; Giovannoni et al., 2017). However, the role that organic acids play during this process is currently not fully understood. Are the complex organic acid profile changes over the course of fruit development simply a consequence of the process or do they play an active role in the sequence of events leading to fruit maturation? Here, we provide an overview of the latest discoveries and suggest future directions regarding organic acids metabolism during fruit development and ripening. We first discuss the general roles of organic acids during fruit maturation, we then focus on the metabolic behavior of those compounds and their relationship with both sugars and hormones during fruit development. Finally, we highlight the importance in studying organic acid metabolism during both fruit development and fruit ripening on different fruits and outline strategies to improve both qualitative and quantitative traits of crop fruits.

## The Functional Diversity of Organic Acids: More Than Simple Intermediaries?

**FIGURE 1:**
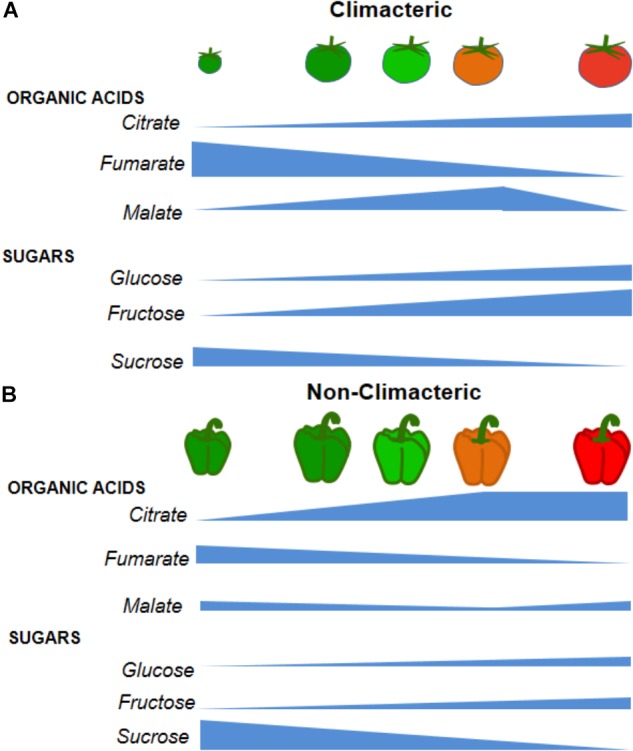
Comparative metabolite accumulation during fruit growth in two significant examples of climacteric (tomato, *Solanum lycopersicum*) and non-climacteric (pepper, *Capsicum* spp.) species. **(A)** Organic acids and sugars changes that occur in climacteric fruits during different stages of development and ripening using tomato fruit as a model of climacteric fruit. The concentration of each metabolite is dependent on the metabolic intensity and the time of development of the fruit with major changes during the climacteric peak phase (approximately 35 days after anthesis). **(B)** Organic acids and sugar changes that occur in non-climacteric fruit during different stages of development and ripening using pepper as a model. Although lacking a climacteric peak, fruits considered non-climacteric present metabolite profile alterations during maturation, but with lower intensities than in climacteric fruits.

During fruit development, organic acids levels are usually inversely related to sugar levels. As such, during maturation and ripening, sugars accumulate, mainly due to sugar import or from starch degradation, whereas organic acids that accumulated in young fruits strongly decrease (Carrari et al., 2006; Carrari and Fernie, 2006; Fait et al., 2008; Mounet et al., 2009; Beauvoit et al., 2018). Malate and citrate are considered the most abundant organic acids present in both climacteric and non-climacteric ripe fruits (Figure 1). Particularly, malate accumulation and degradation occur differently in climacteric and non-climacteric fruits (Figure 1). Whilst some climacteric fruits use malate as a substrate during the respiratory burst, non-climacteric continue accumulating malate throughout ripening (Cherian et al., 2014). Interestingly, citrate levels are largely decreased during the ripening process followed by decreases in malate as a respiratory substrate after the climacteric peak in papaya fruits (Manrique and Lajolo, 2004; Cosme Silva et al., 2017) (Table 2). Equally, during ripening of the non-climacteric orange and lemon fruits there is a decline in titratable acidity, mostly due to the catabolism of citrate (Iglesias et al., 2007; Agrumes et al., 2018) (Table 2). In fact, the metabolism and accumulation of organic acids in fruits are under both genetic and environmental control (Etienne et al., 2013). Moreover, through principal component analysis, the existence of a highly conserved change in the dynamics of metabolic processes during fruit development and ripening across species belonging to climacteric and non-climacteric groups has been recently demonstrated (Klie et al., 2014). Therefore, enhancing our current understanding of these factors and their interactions is of pivotal importance for fruit quality improvement.

**Table 2:** Metabolic behavior of different sugars and organic acids present in the mesocarp of different fruit during growth and ripening under optimal growth conditions.

	Sugars			Organic Acids			
Fruits	Glucose	Fructose	Sucrose	Citrate	Malate	Fumarate	Reference
Apple	Increase	Increase	Increase	–	Increase	–	Ackermann et al., 1992; Wu et al., 2007; Zhang et al., 2010
Banana	Increase	Increase	Decrease	–	Increase	–	Morvai and Molnár-Perl, 1992
Grape	–	–	–	–	Increase	–	Sweetman et al., 2009
Guava	Increase	No change	Traces	Increase	Traces	–	Bashir and Abu-Goukh, 2003; Jain et al., 2003; Batista Silva et al., 2018
Kiwifruit	–	–	–	–	Increase	–	Walton and Jong, 1990; Cui-Cui et al., 2018
Lemon	Increase	Increase	Decrease	Increase	No change	–	Albertini et al., 2006
Lime	No change	Increase	increase	Increase	Increase	–	Albertini et al., 2006
Melons	Increase	Increase	Increase	–	–	–	Seymour and McGlasson, 1993; Karaman et al., 2018
Orange	Increase	Increase	Increase	Increase	No change	–	Albertini et al., 2006; Guo et al., 2016; Zhou et al., 2018
Papaya	No change	No change	Traces	Decrease	–	–	González-Aguilar et al., 2003; Souza et al., 2014
Peach	Increase	Increase	Decrease	Decrease	Increase	–	Cirilli et al., 2016
Pineapple	Increase	Increase	Few changes	Increase	Few changes	–	Saradhuldhat and Paull, 2007; Luengwilai et al., 2018
Plum	No change	Increase	Decrease	Increase	Increase	Increase	Famiani et al., 2012
Strawberry	Increase	Increase	Increase	Increase	Increase	–	Rafeii, 2017; Shanmugam et al., 2017
Tomato	Increase	Increase	No change	–	Increase	Decrease	Carrari and Fernie, 2006; Centeno et al., 2011; Osorio et al., 2012, 2013
Watermelon	Increase	Increase	Increase	–	–	–	Gao et al., 2018

The last decade has witnessed an intensive effort to enhance our understanding of the alternative functions of tricarboxylic acid (TCA) cycle components in addition to their recognized role as energetic intermediaries in plants (Millar et al., 2011). Most studies using transgenic approaches to investigate the role of TCA cycle intermediates, however, have been performed on vegetative organs, such as leaves and roots (Fernie and Martinoia, 2009; Araujo et al., 2012; Zhang and Fernie, 2018). The accumulation of TCA cycle intermediates is highly variable depending on plant tissues, developmental stages and environmental factors, most likely due to its direct link to organic acids export and photosynthesis regulation. However, the complex pathways through which organic acids are metabolized and precise details of how they are regulated *in vivo* remains, to date, insufficiently understood (Sweetlove et al., 2007; Fernie and Martinoia, 2009).

Organic acids can support numerous and diverse functions in plants. For instance, the C3 species Arabidopsis (*Arabidopsis thaliana*), soybean (*Glycine max*) and sunflower (*Helianthus annuus*) can accumulate high levels of fumarate (Fernie et al., 2004; Finkemeier et al., 2013). Higher levels of fumarate have been associated with the supply of carbon skeletons to support growth (Zell et al., 2010). Similarly, malate has not only an important role during photosynthesis in CAM and C4 plants (Fernie and Martinoia, 2009; Zell et al., 2010), but has also been associated with stomata regulation (Medeiros et al., 2016, 2017). Remarkably, malate and fumarate levels show similar diurnal changes to those of carbohydrates in some C3 plants, wherein they increase during the day and decrease during the night, suggesting that these organic acids can also function as transient carbon storage molecules (Fahnenstich et al., 2007). This fact apart, the contribution of organic acids to metabolic processes affecting fruit development and fruit quality remains to be elucidated. Dissecting these mechanisms is required to fully understand the key components underlying organic acid metabolism on energetic processes in fruit growth and development.

The function of TCA cycle intermediates have been extensively demonstrated in diverse aspects of plant growth (Nunes-Nesi et al., 2008; Araújo et al., 2014b) and in response to stress conditions (Sweetlove et al., 2010; Nunes-Nesi et al., 2014). Additionally, signaling functions have also been recently demonstrated for different TCA cycle intermediates from human (Yang et al., 2012) to plant (Finkemeier et al., 2013). Thus, citrate (Wellen et al., 2009), fumarate (Yang et al., 2012) and succinate are all involved in signaling in animal cells whereas also citrate (Gray et al., 2004) malate (Gray et al., 2004; Geigenberger and Fernie, 2014) and 2-oxoglutarate (Huergo and Dixon, 2015) were all recognized to play signaling functions in plants. Remarkably, the mode of action of these metabolites within the signaling network in which they are involved is variable (Wellen et al., 2009). For instance, it has been demonstrated that citrate regulates the expression levels of genes related to alternative respiratory pathways in both tobacco (*Nicotiana tabacum*) and Arabidopsis (Gray et al., 2004; Clifton et al., 2005). In addition, citrate, malate, and 2-oxoglutarate can affect nitrogen assimilation by controlling the abundance of Nitrate Reductase (NR) transcripts in tobacco (Müller et al., 2001). It is reasonable to assume, therefore, that TCA cycle intermediates are good candidates to play signaling roles in angiosperm fruit development as well as during fruit maturation and ripening.

Transgenic tomato plants with differential expression of all genes encoding TCA cycle enzymes have been generated and characterized (Nunes-Nesi et al., 2013). Collectively, this has allowed the generation of a thorough set of plant lines in which the activity of enzymes in the pathway are progressively decreased. The characterization of these plants has provided advances in our knowledge regarding the TCA cycle metabolic connections with other metabolic pathways (Fernie et al., 2004; Sweetlove et al., 2010). Indeed, these studies provided compelling evidence of the distribution of control in the plant TCA cycle. Moreover, they have also demonstrated that organic acids play important functions in the control of several important processes in connection with mitochondrial metabolism, including photosynthesis (Nunes-Nesi et al., 2005), carbon to nitrogen metabolism (Araujo et al., 2008), and redox balance (Igamberdiev and Bykova, 2018).

The signaling importance of TCA cycle intermediates might also rely on how exactly plant metabolism is reprogrammed following changes in their levels. For instance, reductions on the expression of aconitase (ACO) (Carrari et al., 2003) and malate dehydrogenase (MDH) resulted in reduction in both fruit size and yield. Years later, Nunes-Nesi et al. (2007) used transgenic tomato plants deficient in the mitochondrial fumarase activity (FUM) to show a strong effect on photosynthesis caused by impairments in the stomatal function followed by a subsequently reduced TCA cycle flux, affecting carbohydrate and organic acid oxidation at the whole plant level. Tomato plants with reduced expression of citrate synthase (CS) (Sienkiewicz-Porzucek et al., 2008), NAD-dependent isocitrate dehydrogenase (NAD-ICDH) (Sienkiewicz-Porzucek et al., 2010), cytosolic NADP-dependent Isocitrate dehydrogenase (NADP-ICDH) (Sulpice et al., 2010) and 2-oxoglutarate dehydrogenase (OGDH) (Araújo et al., 2012) showed no differences in either carbon assimilation or fruit yield. In contrast, a mild reduction in mitochondrial NAD-ICDH, as well as NADP-ICDH activity in antisense transgenic lines resulted in altered nitrate assimilation and pigmentation and amino acids contents, coupled with reduced fruit diameter and fresh weight, probably associated to source:sink alterations (Sienkiewicz-Porzucek et al., 2010; Sulpice et al., 2010). Additionally, changes in OGDH resulted in early senescence, coupled with significant alterations in metabolites pattern during fruit development (Araújo et al., 2012). Moreover, antisense inhibition of the Iron-Sulfur Subunit of Succinate dehydrogenase (SDH) was associated with enhanced fruit yield (Araújo et al., 2011) whereas SDH and MDH mutant plants were characterized by bigger fruits affecting fruit quality (Nunes-Nesi et al., 2005; Araújo et al., 2011; Centeno et al., 2011).

Collectively, these data provide compelling evidence that the metabolic connections associated with TCA cycle-related organic acids are responsible, at least partially, for such changes and therefore an extensive metabolic reprogramming also occurs in fruits when changes in TCA cycle take place. It seems therefore tempting to speculate that the enzymes of the TCA cycle as potential target to further improve fruit quality. However, further demonstration of the importance of changes in organic acid levels in fruits are required to obtain a full comprehension of this process. This will most likely occur by the genetic manipulation of fruit-specific genes using fruit-specific promoters (Fernandez et al., 2009) to understand fruit maturation effects.

## The Metabolic Behavior of Organic Acids During Fruit Development and Ripening

Fleshy fruit ripening is often characterized by a breakdown of stored carbohydrates to sugars coupled with reductions in acidity alongside with increases in flavor and aroma volatiles (Klee and Giovannoni, 2011; Cherian et al., 2014). It is accepted that organic acids are important in the control of fruit growth via cell expansion through water uptake (Liu et al., 2007). Accordingly, organic acids accumulation during the early stages of fruit development is directly related to the supply of substrates for the maintenance of respiration processes during the development (Seymour et al., 2013). Remarkably, different species including apples, berries, citrus, grape, kiwifruit (*Actinidia deliciosa*), peach, pepper, and tomato, present a highly similar metabolic pattern in which higher organic acids concentration are observed in the first stages of fruit development followed by clear reductions in their levels as maturation progresses (Nardozza et al., 2013; Osorio et al., 2013; Lin et al., 2015). Additionally, quantitative and qualitative variations of organic acids and sugars are usually observed in relation not only to cultivars and genotypes but also during maturation stages, affecting flavor without changes in fruit development and ripening (Xi et al., 2017). Accordingly, as in many other fleshy fruits, malate and citrate are the predominant organic acids identified in ripe peach (*Prunus persica* (L.) Batsch). The accumulation of organic acids is seemingly well regulated during fruit development and is differentially controlled during growth stages (Masia et al., 1992; Moing et al., 2000). By using six peach cDNAs encoding key proteins involved in organic acid metabolism and solute accumulation, Etienne et al. (2002) demonstrated that genes involved in organic acid showed a stronger expression during fruit ripening than during the earlier phases of development. Remarkably, their expression patterns were not necessarily correlated with the changes in organic acid contents (Etienne et al., 2002). The content of organic acids and soluble sugars was evaluated in apricot (*P. armeniaca* L. cv. Harcot), plumcot (plum-apricot hybrid, *P. salicina* × *P. armeniaca* L. cv. Harmony), plum (*P. salicina Lindl*. cv. Formosa), and peach (*P. persica* L. Batsch cv. Jinmi) (Haejin et al., 2014). Notably, organic acids increased mostly during the early stages of fruit growth and decreased until fruits were fully ripen, whereas sucrose, fructose, and glucose, but not sorbitol, increased during fruit development (Haejin et al., 2014).

By investigating a number of grapefruit (*Citrus paradisi*) cultivars produced in Turkey, Kelebek (2010) showed that, in most cases, sucrose was the predominant sugar, followed by fructose and glucose, while citrate was the most abundant organic acid, followed by malate, and that their content increased with ripening. The changes in sugars and organic acid concentrations in six different citrus cultivars Ponkan’ (*C. reticulate*) and ‘Satsuma’ (*C. unshiu*), sweet orange ‘Newhall’ (*C. sinensis*) and ‘Early Gold’ (*C. sinensis*), pummelo ‘HB’ (*C. grandis*) and grapefruit ‘Flame’ (*C. paradise*) was recently analyzed (Zhou et al. (2018). This study has shown that the variations in sugar concentrations of this six citrus cultivars were relatively similar with sucrose as the major sugar component at every stage. Similar results were also previously found (Lin et al., 2015). Kafkas et al. (2007) additionally investigated different strawberry genotypes and found that fructose, the main sugar, increased during ripening. By contrast, the concentration of citrate is variable between genotypes, while the concentration of malate in all genotypes does not change during fruit ripening (Basson et al., 2010). Additionally, a wide comparison of sugars and organic acids content in different genotypes of strawberry, sweet cherry, long mulberry and small mulberry demonstrated that fructose and glucose were the major sugars found in the fruits, while citrate and ascorbate were the predominant organic acids in strawberry and mulberry, and tartaric acid in sweet cherry (Mahmood et al., 2012). In papaya, a climacteric fruit, four cultivars, namely Coorg Honey Dew, Pink Flesh Sweet, Sunrise, and Washington, were characterized according to their chemical composition and sucrose was the predominant sugar in all cultivars (Selvaraj et al., 1982). However, in cv. Washington an increased glucose content was observed 140 days after anthesis in comparison with the others cultivars. Additionally, no changes were observed in organic acids concentration in the different papaya cultivars (Selvaraj et al., 1982). Conversely, seven tomato cultivars were studied in relation to their compositional changes during different ripening stages. It was demonstrated that the sugar content was differentially modified according to the cultivars under different ripening stages with increases in all those seven cultivars most likely due to starch conversion to sugars, whilst fruit acidity was slightly increased in all cultivars (Kaur et al., 2006). Altogether, these studies suggest that sugars and organic acid levels can in fact be highly variable without impacting normal fruit development and ripening.

Over the past decade, much research effort has been devoted to understanding the metabolic behavior of several fleshy fruits ranging from physiology and biochemistry to broad molecular and genetics approaches. Thus, over the following sections we provide a detailed discussion about the organic acids behavior during fruit development as well as its relationship with other important metabolites, paying particular attention to sugars and hormones.

### Organic Acids Versus Sugar Metabolism

The ripening process of fleshy fruits is characterized by coordinated changes in whereby fruit biochemistry and physiology are both drastically altered (Brady, 1987; Giovannoni, 2004; Giovannoni et al., 2017). These changes during ripening are typically variable according to the species and maturation stages as well as in response to stress conditions, often due to changes in secondary metabolism, thereby potentially increasing plant defenses and the concentrations of compounds involved in plant protection (Miller et al., 1998; Giovannoni, 2004; Giovannoni et al., 2017). Nevertheless, the main modifications observed during ripening are associated with color and textural alterations coupled with modifications of sugars, organic acids, and volatile compounds (Giovannoni, 2004). Altogether, such modifications contribute to fruit flavor, especially by adjusting the balance between sugar and organic acids (Chaimanee and Suntornwat, 1994). Accordingly, the major respiratory substrates present in most fruits are carbohydrates and organic acids and both their nature and concentration largely affect organoleptic quality as taste, sight, and smell (Seymour et al., 2013).

Sugar accumulation has been intensely investigated during fruit development in different species under diverse conditions. Throughout ripening the vast majority of fleshy fruits are characterized by increases in sugar contents whereas organic acids decrease (Giovannoni et al., 2017). Citrus species are the exception to this metabolic ‘rule,’ especially at the peak of maturity or ripening. Global transcriptome analysis has suggested that during middle and later stages of citrus fruit development both carbohydrate synthesis and catabolism are mostly down-regulated while sugar transport appears to be rather operative. This can be deduced from the up-regulation of sucrose phosphate synthase (SPS), which in turn is correlated with total soluble solids (TSS) and the up-regulation of citrate synthase (CS) (Cercós et al., 2006; Wang et al., 2017). In parallel with fruit growth, sugars and organic acids are accumulated but in different stages of development. Glucose, fructose and sucrose increase in an exponential manner during cell division phase, reaching stable levels during final growth and ripening process. The content of citrate, the main organic acid found in citric fruits, increases upon cell division stage reaching higher levels in the middle of stage II and decreasing, mostly due to its catabolism, during ripening (Iglesias et al., 2007; Hussain et al., 2017). Both metabolic patterns and concentration are highly variable according to the species. In this vein, accumulation of sucrose, glucose, and fructose during ripening are especially observed in sweet fruits such as apples (Jakopic et al., 2018; Williams and Benkeblia, 2018), litchi (Yang Z. et al., 2013), melons (Burger et al., 2000; Huang et al., 2017), peach (Cirilli et al., 2016), strawberries (Shanmugam et al., 2017), mango (Cosme Silva et al., 2017), papaya (Paull et al., 1999) and watermelons (Liu et al., 2013) (Table 2). In general, sugar accumulation in fruit is directly controlled by increasing the activities of sucrose synthase (Suzy) and SPS (Chen et al., 2004).

Sucrose, glucose, and fructose are the most abundant carbohydrates and widely distributed food components present in plants. Their ratios vary considerably between fruits and, to a lower extent, in the same fruit according to maturation stage (Arena et al., 2013). Notably, the oxidation of such carbohydrates via glycolysis provides substrates for the TCA cycle during cell respiratory processes, contributing not only to the generation of intermediates such as organic acids, but also contributing to cellular energy supply (Osorio et al., 2013). Remarkably, during the climacteric stage, there is a large increase in the rate of substrate oxidation, mediated mainly by mitochondrial oxidases and, as result, there is an increased glycolytic flux. Interestingly, this enhanced flux has been associated with a close relationship between the activities of key glycolytic enzymes such as pyruvate kinase and phosphofructokinase in different fruits including apple, avocado, banana, and tomato (Rhodes and Wooltorton, 1967; Bennett et al., 1987; Beauvoit et al., 2018).

Similarly to sugars, organic acids are also able to support several facets of plant metabolism. Thus, the accumulation of organic acids in plant cells is highly correlated with other metabolic pathways and appears to be under the control of many factors (Lin et al., 2016). Both the organic acid type and its levels are extremely dependent of species, development stages, and the tissue analyzed. Although changes in content of organic acids are strongly fruit-dependent the most abundant organic acids in several fruits are citrate and malate (Romero Rodriguez et al., 1992), both being variable over different stages of fruit development (Table 2). Unlike soluble carbohydrates, which are imported into the fruit as photosynthate, the majority of the organic acids present in fleshy fruits are not imported but rather synthesized *in situ*, mostly from imported sugars from glycolysis mediating starch and cell wall degradation (Etienne et al., 2013). This is in good agreement with findings showing that starch accumulation plays an important role in determining the soluble solids content (°Brix index) of mature fruit, which is directly influenced by the activity of invertases, such as tomato LIN5 (Schaffer and Petreikov, 1997; Vallarino et al., 2017). Therefore, organic acids appear as highly valuable metabolites from a metabolic engineering perspective, once the organic acid-to-sugar ratio defines a range of quality parameters at harvest time in fruits.

By using integrative analyses of metabolomics and transcriptome during fruit ripening in ponkan (*Citrus reticulata*) fruits, it was showed that increases in sugars content are followed by considerable reductions in the content of organic acids (Lin et al., 2015). Perhaps more importantly, it was demonstrated that such behavior might be driven by SPS, asparagine transferases (AST), ATP-citrate lyase and glutamate decarboxylase (GAD) mediating shifts in sucrose metabolism from synthesis to degradation, which was regulated by the balance between SPS and SuSy activity (Lin et al., 2015, 2016). In addition, increased enzyme activity from both glycolysis and the TCA cycle during later maturation were observed, indicating that the flux is somehow changing from sucrose metabolism to organic acid metabolism, with citrate degradation occurring mainly through the gamma-aminobutyric acid (GABA) and acetyl-CoA pathways (Lin et al., 2015, 2016). It was further demonstrated that ponkan fruits under hot air treatment could activate citrate degradation via the GABA shunt especially by modulating aconitase, isocitrate dehydrogenase and glutamate decarboxylase cascade, but not the glycolytic pathway (Chen et al., 2012).

Positive correlation between malate levels and the expression of genes involved in starch synthesis has been observed in pepper (*Capsicum* spp.) fruits, meaning that malate metabolism most likely regulates transitory starch metabolism and that this process is probably conserved between climacteric and non-climacteric fruits (Osorio et al., 2012). Indeed, reduction of the activities of either the mitochondrial malate dehydrogenase (mMDH) or fumarase (FUM) in tomato fruits via targeted antisense approaches have demonstrated the physiological importance of malate metabolism in the activation state of ADP-glucose pyrophosphorylase (AGPase), which is correlated with the accumulation of transitory starch and soluble solids at harvest (Centeno et al., 2011). However, due to the limited amount of information available on the connection between other organic acids metabolism and fruit quality, we cannot rule out a similar role for them during fruit development.

For certain fruits, citrate can be found in considerable concentrations. It is present from 8 to 15% (dry weight basis) in fruits such as strawberries and lemons (*Citrus lemon*). In both lemon and lime (*Citrus aurantifolia*) citrate accumulation can be as high as 8% of the fruit dry weight (Müller et al., 1996). The process involved in the metabolism and accumulation of citrate in mesocarp cells of fruits is under both genetic and environmental control. Several studies using different approaches as transcriptomics (Christelle et al., 2002), proteomics (Katz et al., 2011; Molassiotis et al., 2013) and metabolomics (Guo et al., 2016) have aided in the understanding of the different mechanisms involved in the control of the acidity and the quality of fruits. In addition, different agricultural practices including irrigation, nutrition (Kumar and Kumar, 2007) as well as controlled temperature (Burdon et al., 2007) can also impact the levels of fruit metabolites and as such the ratio between sweetness and acidity. However, there are no clear explanations for the changes observed in both malate and citrate in the cell.

To better understand how citrate metabolism is affected in Ponkan fruits, plants grown at low temperature and water stress in an open field experiment were compared to plants grown in optimal greenhouse conditions (Lin et al., 2016). It was observed that the expression levels of phosphoenolpyruvate carboxylase (PEPC), CS, ACO, and GAD were increased in response to low temperature, but not in water stressed plants compared to control conditions. These results, coupled with the changes in citrate levels under such conditions, indicated that low temperature may be a major factor influencing citrate metabolism during maturation in ponkan fruits. Similarly, it was observed that in sweet orange (*Citrus sinensis*), a non-climacteric fruit, the activities of the enzymes involved in organic acid metabolism including malic enzyme (ME), ICDH, ACO, and alcohol dehydrogenase increased during the first 3 weeks of post-harvest storage. Concomitantly, increased activity of enzymes involved in sugar catabolism such as hexokinase (HXK), Susy, UDPG pyrophosphorylase and PPi-dependent phosphofructokinase was also observed (Echeverria and Valich, 1989). Notably, these enzymes are necessary for organic acid usage and the subsequent oxidization of sugars in harvested sweet oranges (Echeverria and Valich, 1989). Pineapple is characterized by high contents of organic acids (Tables 1, 2), primarily controlled by the activity of key enzymes such as CS, ACO, PEPC, MDH, and ME. In particular, ACO seems to play a major role in modifying the acidity in pineapple (Saradhuldhat and Paull, 2007). Altogether, it seems reasonable to assume that a very tight connection between sugar and organic acid metabolism occurs during fruit development. However, exactly how this metabolic regulation occurs still remains to be elucidated.

Studies with papaya (*Carica papaya*), a typical climacteric fruit, have revealed that the accumulation of sugar, especially sucrose, occurs between 20 and 30 days before physiological maturation. During this stage, there is a significant increase in acid invertase (AI) activity with lower SPS and Susy activities in papaya mesocarp (Zhou and Paull, 2001). It has been also demonstrated that after harvesting there was still sucrose synthesis, and more importantly that the SPS activity is highly correlated with the sucrose content, indicating the importance of this enzyme during the ripening in papaya (Gomez et al., 2002). Although in papaya fruits the main organic acids are citrate, malate, and ascorbate, their accumulation occurs to relatively low concentrations (de Oliveira and Vitória, 2011). Particularly, citrate and malate contents are reduced over the course of ripening (for details see Table 2) (Brekke et al., 1971; Souza et al., 2014; Silva et al., 2015).

Analysis of sugars and organic acids content during the development of peach (*Prunus persica*) fruits showed that malate, quinate and shikimate concentrations were high at the beginning but declined at the end of fruit development (Wu et al., 2005). Thus, citrate concentration was maximal in immature fruits, whereas increased sugars concentration, mainly sucrose, occurred in mature fruits. Interestingly, during peach ripening, sucrose degradation was accompanied by an increase of glucose and fructose levels coupled with distinct regulation of transcripts encoding neutral invertases (NI), indicating differential or non-redundant functions of each putative NI isoform in peach (Borsani et al., 2009). Enzymes such as NI and PEPC were identified as important components of the carbon metabolism operating during peach post-harvest ripening (Borsani et al., 2009).

Malate is the predominant organic acid in many fruits, including both climacteric and non-climacteric fruits such as plum (*Prunus salicina*), banana, tomato, grape and apple (Morvai and Molnár-Perl, 1992; Sweetman et al., 2009; Centeno et al., 2011). Citrate is the predominant organic acid in citric fruits like oranges, lime and lemon (Albertini et al., 2006). Notably, citrate is also found as the main organic acid in grape, guava, papaya, pineapple and strawberry as show in Table 1 (Clements, 1964; Brekke et al., 1971; Jain et al., 2003; Batista Silva et al., 2018). Nevertheless, the accumulation and degradation of organic acids are not directly associated with respiratory and climacteric characteristics of the fruit. Thus, it is known that some climacteric fruits such as tomato appear to utilize malate during the respiratory burst (Goodenough et al., 1985). By contrast, banana and mango (*Mangifera indica*) continue to accumulate malate throughout ripening, even at the climacteric stage (Selvaraj and Kumar, 1989), whilst non-climacteric fruits also display widely varying malate accumulation and degradation (Sweetman et al., 2009). In addition, the accumulation of malate and citrate is seemingly a result of close interaction between metabolism and vacuolar storage and is also controlled by several environmental factors that affect the acidity of fleshy fruit by acting on various cellular mechanisms (Etienne et al. (2013). Taken together, the close relationship between organic acids and sugars metabolism during ripening seems to be a broadly connected factor that contribute in every way to the improvement of quality and flavor during fruit ripening. For this reason, it seems clear that a more in-depth understanding of the assessment of sugars and organic acids content and the use of genetic and agricultural tools capable of changing this relationship is of pivotal interest to food experts and researchers. Collectively, these results indicate that alterations in genes involved in the organic acids metabolism can determine the quality and extension of shelf-life of non-climacteric fruits, which have their physiological changes reduced after being detached from the plant.

### Organic Acids Versus Hormonal Control of Fruit Development and Ripening

**FIGURE 2:**
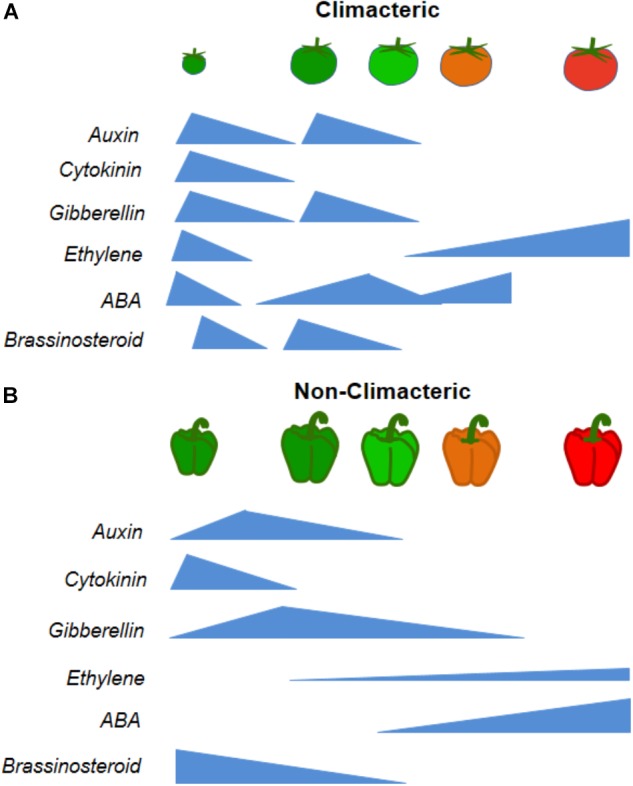
Hormonal pattern changes of two significant examples of climacteric *(Solanum lycopersicum)* or non-climacteric (*Capsicum* spp.) during fruit development and ripening. **(A)** Differential hormones concentrations in tomato fruit during development: increases in auxin, cytokinin, gibberellin, and brassinosteroids at fruit set, followed by increases in auxin, cytokinin and gibberellin at fruit growth until maturation, which have their levels drastically reduced, with increases in ethylene and abscisic acid (ABA). **(B)** Differential hormonal patterns in non-climacteric fruit during development show increases in auxin, cytokinin and gibberellin during fruit set and growth, followed by reductions along the maturation phase, with increases in ABA and brassinosteroids, and few changes in ethylene.

Although hormones have been extensively studied as signaling molecules involved in different aspects of plant life cycle, it has been only recently that research has focused on understanding of their action during fruit development as well as in controlling sugars and organic acids metabolism in fruits during the ripening process (Figure 2) (McAtee et al., 2013; Karlova et al., 2014; Kumar et al., 2014). Accordingly, it has been demonstrated that hormones affect sugars and starch metabolism and that they can extend post-harvest life (Sagar et al., 2013a; Bastías et al., 2014; Karlova et al., 2014). However, there is a scarce literature available on the hormonal control of starch hydrolysis and the resulting sugar accumulation coupled with mitochondrial respiration (McAtee et al., 2013; Kumar et al., 2014). This fact apart, both the maturation and ripening have been associated in a number of studies to metabolic alterations involved with multiple genetic and biochemical pathways (Osorio et al., 2013). Although these changes have been observed in the context of responses to hormones (e.g., ethylene and ABA), the link between hormonal control and metabolite accumulation remains rather limited (Giovannoni, 2004; McAtee et al., 2013; Giovannoni et al., 2017).

The advent of ‘omics’ approaches has enabled significant progress in the characterization of hormone responses in fruits in general (Osorio et al., 2011). In addition, one important aspect of fruit development is the modulation of its metabolism, mainly driven by changes in sugars, organic acids and secondary metabolites immediately after fruit setting and partially recovering during or after ripening (Carrari et al., 2006). According to (Bapat et al., 2010), in climacteric fruit such as tomatoes, papaya, peaches, banana, apples, melon and other, ethylene synthesis plays a predominant role during ripening, and this still remains as one of the most studied hormones (Figure 2A). On the other hand, in non-climacteric fruits the respiratory burst and rise in ethylene production are not evident. It is widely accepted that although no single ‘master controller’ is able to control the ripening in non-climacteric fruits. Increased levels of different hormones like ethylene, abscisic acid (ABA), and brassinosteroids (BRs) have been suggested to promote ripening through complex interactions, whereas auxin delays some ripening associated process in those fruits (Figure 2B) (Fortes et al., 2015). Thus, we will now briefly discuss how changes in the main hormones involved in the process of formation and ripening of fruits impact organic acid metabolism, controlling fruit composition via crosstalk with other hormones or by themselves.

Ethylene has been shown to control many ripening-associated metabolic pathways. It is involved not only in the expression of senescence associated genes and defense signaling, but also in fruit ripening, where the autocatalytic ethylene production leads to changes in cell wall metabolism, carotenoid accumulation, chlorophyll degradation, synthesis of volatiles compounds, and modulation of sugars and acids contents (Giovannoni, 2001; Alexander and Grierson, 2002; Osorio et al., 2013; Farcuh et al., 2018).

The importance of ethylene in the production of aroma volatiles has been also genetically demonstrated by the antisense suppression of ethylene production, which resulted in strong inhibition of aroma in melon (*Cucumis melo* L.) fruits (Ayub et al., 1996). The ripening of the climacteric fruit peach is largely controlled by ethylene and thus increase ethylene production leads to enhanced respiration coupled with changes in both chemical composition and physical characteristics of the fruit (Paul et al., 2012). Furthermore, enhanced ethylene biosynthesis are accompanied by increased levels of citrate, malate and glucose and fructose but decreased sorbitol and sucrose levels following harvesting (Borsani et al., 2009).

It has been also shown that ethylene is involved in an organ-specific manner in strawberry fruit ripening by differentially controlling the levels of amino acids, glucose, and fructose, as well as citrate and malate in the achene and the receptacle (Merchante et al., 2013). Strawberry plants with altered sensitivity to ethylene were used to unravel its role during fruit ripening process, as well as to further enhance our understanding of the modulation of metabolic pathways (Merchante et al., 2013). Similarly, it has been demonstrated that in grape, a non-climacteric fruit, ethylene seems to be also required for berry development and ripening (Chervin et al., 2004). Indeed, it has been suggested that ethylene could be triggering the onset of ripening. In fact, Ethylene Responsive Factor (*VviERF045)* from grape affects a range of different processes including, photosynthetic capacity, secondary metabolism, expression of key genes related to changes in epidermis and cuticle of the berry, cell expansion, as well as activation of several defense related genes (Leida et al., 2016).

Recent examples of cross-talk among different hormones have revealed a highly complex interplay of signals during grape development and ripening (Fortes et al., 2015). Accordingly, in non-climacteric fruits the responses of ethylene seem to be associated, via highly specific cross-talk, with other hormones such as ABA, auxin (Davies et al., 1997; McAtee et al., 2013) and BRs (Symons et al., 2006), all of which are known to play a functional role in grape berry ripening. Non-climacteric fruits may also display climacteric-like behavior following harvest (Katz et al., 2004). Similarly, differential expression of component of the ethylene-signaling pathway have been also observed in several non-climacteric fruits including citrus and grape (Tesniere et al., 2004; Trainotti et al., 2005).

The participation of ethylene in ripening has been extensively investigated in tomato fruits using several mutant that drastically affect the ripening processes of tomatoes fruits. For instance, *ripening inhibitor* (*rin*), *non-ripening* (*nor*), *green ripe* (*Gr*), *green-flesh* (*gf*), *colorless non-ripening* (*Cnr*), *never ripe* (*Nr*), *high pigment 1 (hp1*), *high pigment 2* (*hp2*), and *dark green* (*dg*) have been investigated in the context of ripening (Lanahan et al., 1994; Mustilli et al., 1999; Vrebalov et al., 2002; Levin et al., 2003).

Although the use of such mutants has clearly provided significant insights on the respective functional roles and also hierarchical regulation based on each gene (Giovannoni, 2004), the complete understanding of the ripening regulatory network remains rather fragmented. Nevertheless, by analyzing three dominant ripening mutants of tomato, *nor, rin, and Nr*, along the developmental and ripening periods it was possible to identify very strong correlations between ripening-associated transcripts and specific metabolite groups, such as organic acids from the TCA cycle, sugars, and cell wall-related metabolites, such as lipoxygenase, pectate lyase and poligalacturonase (PG) underlining the importance of these metabolic pathways during fruit ripening (Carrari et al., 2006; Centeno et al., 2011; Osorio et al., 2011). Organic acids, including the two TCA cycle intermediates malate and citrate, were strongly affected across ripening, suggesting that organic acids are regulated at the transcriptional level in climacteric fruit. Importantly, malate plays a crucial role in transitory starch metabolism in normal tomato fruit development and ripening and it seems that its regulation is also conserved in non-climacteric fruits (Centeno et al., 2011; Osorio et al., 2011, 2012, 2013). Additionally, in strawberry fruit organic acids including succinate, fumarate and 2-oxoglutarate displayed substantial changes during ripening, associated with a heavy demand for carbon skeleton components (Fait et al., 2008). In pepper fruit, citrate, dehydroascorbate, and malate are highly correlated to genes associated with starch and cell wall pathways as well as protein degradation, suggesting the importance of these organic acids during pepper fruit development and ripening (Osorio et al., 2012). Altogether, these results underlie the pivotal significance of the metabolic pathways associated with sugars and organic acids, revealing also multiple ethylene-associated events that occurs during climacteric and non-climacteric fruit ripening. It is important to mention that ethylene does not act regulating ripening alone but it rather works likely in conjunction with others phytohormones such as auxin, ABA, and cytokinin (McAtee et al., 2013; Kumar et al., 2014).

Future studies should therefore explore the hormone signaling network by combining model plant based knowledge on the molecular mechanisms involved in hormone signaling and the association with available genome information of other plant species. In apple fruit, the main organic acid is malate (Table 1). By using the 1-methylcyclopropene (1-MCP) (inhibitor of ethylene perception) it has recently been shown that ethylene is involved in the regulation of the levels of organic acid once this compound delayed the reduction of malate and citrate content during ripening (Lu et al., 2013).

In fruits with lower ethylene requirement to ripen, ABA appears to have a crucial role given its increase following ripening process (Setha, 2012). In strawberry, a non-climacteric fruit model, the effect of ABA has been investigated (Jiang and Joyce, 2003) demonstrating that endogenous ABA may play a role in changes of fruit color during ripening via an up-regulation of both ethylene production and phenylalanine ammonia-lyase (PAL) activity. In good agreement, compelling evidence suggest that exogenous ABA can significantly accelerate strawberry fruit ripening, most likely by the down regulation of 9-*cis*-epoxycarotenoid dioxygenase gene (*FaNCED1*) as demonstrated by virus-induced gene silencing (VIGS) leading to decreased content of ABA that can significantly retard the ripening (Jia et al., 2011). The *FaNCED1* is a predominant contributor to ABA accumulation during fruit ripening and it has been also evidenced that soluble sugars, especially sucrose, may act as a promoter to trigger ABA accumulation (Jia et al., 2011). The interaction between sugar and ABA has been recently reviewed by (Li et al., 2011), suggesting a core mechanism involved in the regulation of non-climacteric fruit ripening. Remarkable, reduced expression of *NCED* in strawberry resulted in delayed fruit maturation with changes in several metabolites such as organic acids and sugars implicating ABA in the control of fruit quality.

In climacteric fruits such as tomato and banana the levels of ABA increased before an increase in ethylene (McAtee et al., 2013). Remarkably, ABA signaling may also impact different aspects of fruit maturation (Sun et al., 2012). Exogenous ABA treatment consequently increase the ABA content in both flesh fruits and seeds, triggering ethylene biosynthesis by the up regulation of ACS and ACO expression and therefore inducing fruit ripening (Zhang et al., 2009). It has been also suggested that *LeNCED1*, which initiates ABA biosynthesis at the onset of fruit ripening, can be the original inductor of ABA accumulation and might play a key role not only in the ripening process but also during the senescence of tomato fruits (Zhang et al., 2009). The suppression of the gene encoding *NCED1* resulted in a down regulation of several other genes involved in ripening process including the cell wall related PG and pectinmethylesterase (PME), which can also contribute to changes in TCA cycle intermediates (Sun et al., 2012). Although those studies collectively provided evidence that ABA is involved in fruit maturation, it still remains unclear whether ABA acts directly or via altering ethylene levels, given the already well established cross-talk between those hormones.

Overexpression of ABA-responsive elements *SlAREB1* in tomato, resulted in increased content of organic acids (e.g., citrate and malate), hexoses, hexose-phosphates, and amino acids in immature green, mature green, and red ripe fruits (Bastías et al., 2014). These modifications correlated with an up-regulation of genes encoding enzymes involved in carbohydrate and amino acid metabolism suggesting a possible role for this transcription factor in the regulation of fruit organoleptic properties (Bastías et al., 2014). Whether modification of the expression of other enzymes of the TCA cycle involved in the synthesis of organic acids and amino acids are affected by *SlAREB1* remains to be determined. In tomato fruits, organic acids are a crucial quality determinant during ripening process and flavor, and correlate with the expression of genes associated with ethylene and cell wall metabolism-related pathways (Carrari et al., 2006; Carrari and Fernie, 2006; Osorio et al., 2012). Further studies are clearly required to elucidate the real mechanism connecting ABA and fruit ripening as well as metabolites changes and fruit quality.

It is currently accepted that auxin participates in various processes ranging from fruit formation to ripening, mainly via a crosstalk between gibberellins and ethylene (McAtee et al., 2013). In fact, auxin coordinates the ethylene synthesis consequently the ripening process (Li et al., 2016). Accordingly, genes related to carotenoid metabolism, cell degradation, and energy metabolism were strongly down-regulated by exogenous auxin further impacting tomato ripening (Su et al., 2015). Recently, RNA-Seq analysis of tomato fruit following exogenous auxin application has shown that several genes involved in the TCA cycle and oxidative phosphorylation pathway were significantly down-regulated indicating that auxin affects fruit ripening by impacting mainly fruit respiration rate (Li et al., 2016). Moreover, auxin-treated fruits were characterized by increased levels of citrate, succinate and malate which indicate that auxin application seems to enhance fruit acidity (Li et al., 2017). Furthermore, exogenous auxin altered the expression patterns of ethylene and auxin signaling-related genes during ripening, suggesting a significant crosstalk between these two hormones during tomato ripening (Li et al., 2016). Recently, an important role for auxin during ripening as a modulator of the levels of sugar and organic acids has been demonstrated in tomato fruits (Sagar et al., 2013a; Hao et al., 2016; Li et al., 2017).

Recent studies have also demonstrated that loss or gain of function of several auxin response genes, such as *SlARF4*, *SlARF2a*, *SlIAA17* and *SlIAA27*, leads to conspicuous changes in fruit pigment accumulation, sugar content, starch accumulation, phenylpropanoids component, organic acids contents and other fruit quality attributes (Bassa et al., 2013; Sagar et al., 2013a,b; Hao et al., 2015; Su et al., 2015). By using a transcriptome analysis approach Li et al. (2016) have suggested that exogenous auxin retards tomato ripening process and interferes on the normal expression patterns of many genes involved in metabolic pathways. More recently, Li et al. (2017) analyzed the metabolic changes following exogenous auxin showing that besides metabolites such as sugars and amino acids, a total of nine organic acids were detected in tomato fruits under different developmental stages. Briefly, higher contents of succinate and ascorbate when compared with control samples were observed 10 days after auxin treatment. Notably, auxin seems to affect citrate levels keeping it higher than in control fruits at the end of ripening, indicating that auxin application might increase fruit acidity, affecting sour taste of fruit (Li et al., 2017).

Over the last decades have witnessed the characterization of numerous mutants for synthesis or signaling of several hormones in different model species such as Arabidopsis (Gazzarrini and McCourt, 2003), tomato (Carvalho et al., 2011), and rice (Yang D.-L. et al., 2013). This resource, coupled with the integration of transcriptomics and metabolomics approaches, has greatly enhanced our understanding of the molecular and biochemical events associated with ripening in both climacteric and non-climacteric fruits. However, despite our current understanding of how organic acid metabolism is associated with hormones metabolism, the exact mechanisms underlying their interaction during fruit ripening clearly require further elucidation.

The role of GA is well established during fruit-set and fruit development, controlling the cell expansion and it has been revisited recently (McAtee et al., 2013; Obroucheva, 2014). However, there is evidence that GA can delay tomato fruit ripening by preventing some of the changes triggered by ethylene. Unfortunately, relatively little work is current available directly connecting gibberellins and metabolic changes in fruits. Nevertheless, some advances were observed on this theme. Accordingly, the impacts of GAs on primary metabolism have been also previously demonstrated in tomato plants with reduced levels of the TCA cycle enzyme 2-OGDH (Araújo et al., 2014a). In the same vein, it has been observed that gibberellic acid (GA3) causes ripening delay in citrus (Biale, 1978) and mango fruits, reducing the ascorbate content (Kader, 2008). In strawberry, GA3 showed an inhibitory effect on fruit ripening, evidenced by a decrease in the respiratory activity (Martínez et al., 1994). GAs seems to affect the primary metabolism mediated by changes in 2-oxoglutarate, thus linking TCA cycle function with amino acid, glucosinolate, flavonoid, alkaloid, and gibberellin biosynthesis (Araújo et al., 2014a). Defining the precise nature of the interaction between organic acids coupled with the GA-mediated regulation of fruit clearly remains an exciting topic for future research.

Due to the multifunctionality of BRs, more attention has been given to their association with fruit ripening recently. BRs plays an important regulatory role in various physiological processes, including growth, seed germination, flowering, changes in enzymatic activities, and fruit set (Clouse and Sasse, 1998; Khripach et al., 1998; Sánchez-Rodríguez et al., 2017) and has been recently associated with fruit ripening. Exogenous BRs analogs on endive (*Cichorium endivia* L.) play an important role in increasing the contents of the organic acids such as citrate, oxaloacetate and succinate (Mario et al., 2013). Mazorra Morales et al. (2014) showed the interconnection between BRs and ethylene in the regulation of the mitochondrial electron transport chain in post-harvest of papaya fruits. The authors showed that exogenous BRs application affect the AOX-dependent electron transport, which is antagonized by ethylene, suggesting that, BRs and ethylene act antagonistically regulating the AOX capacity during papaya ripening. The role of BR application has been also investigated in strawberry, suggesting it as an important molecule to improve qualities traits, mainly by increasing soluble solid contents, inducing sugar and organic acids content, as well as the production of secondary metabolites such as anthocyanin and phenolic compounds (Rafeii, 2017). The role of BRs during fruit ripening has been also investigated in various fruits such as tomato (Vidya Vardhini and Rao, 2002), grapes (Symons et al., 2006), papaya (Mazorra et al., 2013), strawberry (Mohammadrezakhani et al., 2016), and mango (Zaharah et al., 2012). Notably, it is directly related with an extensive crosstalk with ethylene levels, affecting numerous processes. However, relatively few studies have clearly demonstrated the impacts of this phytohormone on primary metabolism and specifically at the organic acids, although it is possible to observe that their content usually increase in presence of exogenous BRs, which can be an interesting avenue for research.

Polyamines (PAs), another group of signaling molecules, has been extensively studied in recent years. PAs are small aliphatic amines with an important role in plant growth process including fruit ripening and (Walden et al., 1997; Guo et al., 2018; Wuddineh et al., 2018). In plants, PAs are initially converted from glutamate, a key amino acid involved in N assimilation, to putrescine (Put), then converted to spermidine (Spd) and, in the end, to spermine (Spm) by the action of Spd synthase (SPDS) and Spm synthase (SPMS) being further decarboxylated to *S*-adenosyl-L-methionine (dcSAM) generating SAM as a reaction product which is catalyzed by SAM decarboxylase (SAMDC) (Wen-Wei et al., 2006; Michael, 2016; Guo et al., 2018). SAM is a common precursor for both PA and ethylene biosynthesis but their physiological functions are distinct at times and can be antagonist mainly during senescence (Pandey et al., 2000).

Accordingly, Gupta et al. (2013) showed that silencing of 1-aminopropane-1-carboxylate synthase gene (*SlACS*) delays ripening simultaneously improving fruit quality in tomato and increasing the PAs levels associated with down-regulation of cell wall hydrolyses. Notably, still in tomato fruit, PAs has been identified as a great contributor of fruit ripening mainly associated with the activity of both ornithine decarboxylase (ODC) and arginine decarboxylase (ADC) (Rastogi and Davies, 1990, 1991). In this vein, Pandey et al. (2015) demonstrated that the overexpression of ODC triggers the biosynthesis of Put, Spd and SPM which, in its turn, inhibits ethylene production delaying fruit ripening, but enhances tomato fruit quality traits. Additionally, overexpression of *SPDS* in tomato promote fruit ripening, increasing sugars content, as well as lycopene coupled with ethylene production (Neily et al., 2011). In grape fruits, PAs also have an important role in the aroma development (Fortes et al., 2015), while in peach it plays a key role in fruit firmness and soluble sugar content followed by an abrupt decreased in Put during post-harvest (Liu et al., 2006). Application of exogenous Spr in peach fruits reduced ripening by impacting ethylene and auxin metabolism and signaling (Patrizia et al., 2012). Remarkably, polyamines are reported to be important molecules involved in strawberry ripening (Tilak and Raymond, 1996). Recently, demonstrated that PAs, especially spermine (Spm), regulate strawberry fruit ripening in an ABA-dominated, IAA-participating and ethylene-coordinated manner controlling several physiological parameters, including firmness, and the content of anthocyanin, sugar, polyamine, auxin (IAA), abscisic acid (ABA), as well as ethylene emission. Notably, these changes are coupled with alterations in *FaSAMDC* expression which can promote and inhibit ripening (Guo et al., 2018). PAs play also important functions in several others fruits in a manner which may generate controversial conclusions, thus it is important to mention that, more studies are required to further understand the significance and roles of PAs in dry and fleshy fruit development.

Lastly, but not least important, very few studies have demonstrated the association between salicylic acid (SA) and the maintenance of fruit quality during post-harvest. Sweet cherry treated with exogenous SA revealed an effective and environmentally friendly tool to maintain fruit quality during storage associated with the maintenance of the sugar and organic acid content in the fruit as well as with enhancements of both the concentration of bioactive compounds concentration and the antioxidant activity (Giménez et al., 2016). Interestingly, SA also culminated with delays on ripening process in banana (Srivastava and Dwivedi, 2000), sweet cherry (Yao and Tian, 2005), and kiwifruit. In all the above mentioned results, SA seems to act inhibiting fruit ripening, mainly by reducing not only the respiratory rate but also sugar and total acid content. It seems reasonable to assume that coupling the application of such hormones or chemical compounds with both molecular and metabolic analysis in order to provide information concerning the role of hormones in the regulation of fruit taste should greatly facilitate advances in our understanding of the metabolic control mediated by hormones in fruits.

## Future Avenues for Unveiling the Role of Organic Acids Metabolism During Fruit Development and Ripening

Although changes in the levels of organic acids are unequivocally important during fruit ripening, it seems necessary to study post-harvest physiology in more realistic environments, which means creating links with companies involved in fruit storage and transport and breaking down the variables that affect organic acid content and other important traits. The understanding of the primary metabolism in fruit is directly connected with fruit quality and seems to be an obvious target for future improvement, however, the complicating factor in this approach is that the metabolism is very dynamic over fruit development and changes are considerable throughout the fruit growth until ripening with many signaling process. Nevertheless, emerging tools can nowadays provide the opportunity to turn this information into a mechanistic understanding of fruit quality, and ultimately to design better fruits in which studding primary metabolism alongside with modeling tools can proved novel information into a mechanistic understanding to mainly develop better fruits (Beauvoit et al., 2018). Additionally, the attention has turned to synthetic biology approaches, mainly by multigene engineering toward multi-gene interventions as recently reviewed elsewhere (Kopka and Fernie, 2018). In parallel, the adoption of synthetic biology may directly provide more effective connections that would circumvent problems associated with feedback regulation of the plants native enzymes and the interactions between TCA cycle and many other processes in the plant could be further expanded as the previous demonstration of signaling function (Gilliham and Tyerman, 2016).

Genetic engineering technologies such as CRISPR/Cas9 could be used to specifically edit the sequence (Čermák et al., 2017; Zsögön et al., 2017) or alter the transcriptional rates (Lowder et al., 2018) of specific genes. Multiplex approaches, targeting various genes simultaneously, are ideally suitable to better understand genetic networks and their interactions (Lowder et al., 2015; Jin et al., 2016). The suitability of this approach has recently been demonstrated in fleshy fruit species such as tomato (Hashimoto et al., 2018) and kiwifruit (Zupeng et al., 2018). The fast pace of advance and improvement in genome engineering techniques, such as the recent introduction of improved endonucleases (Moreno-Mateos et al., 2017; Li et al., 2018) or even single-nucleotide base editing without DNA cleavage (Komor et al., 2016; Gaudelli et al., 2017) suggest that highly efficient genome manipulation tools will soon be available to dissect the complex genetic network involved in fruit maturation control.

Finally, to increase our understanding of the quality and how specific compounds can be changed to improve the ratio between acidity and sugar in fleshy fruits, it seems that coupling integrative approaches (omics) with systems biology is necessary. This would allow the generation of plants, or better fruits, more adapted to stress conditions. Importantly, it is a general opinion that a fruit is a reflection of the conditions to which the plant has been exposed during its development (Poiroux-Gonord et al., 2010). In summary, to increase our knowledge on metabolism during fruit development and the pivotal importance that organic acid metabolism plays on it further research is clearly required.

Considering the importance of amino acids profile during fruit ripening, such as glutamate, the major amino acid of ripe fruits, the usage of such tools could facilitate investigation and simultaneously increasing quality or even extending fruit shelf life, mainly with higher reparatory rate and reduced postharvest time. Teasing out the connections of organic acid metabolism with a hormones may help us understand the real contribution of each hormone on central metabolism. Moreover, rational bioengineering of plants with modified levels of organic acid would also benefit from an increased knowledge of the biochemical regulations and connections inherent to the metabolism of organic acids. The development of plants with altered organic acid composition in fruits should also take into consideration that this pathway is tightly connected with several other aspects of plant metabolism. As such, changes in organic acid metabolism within fruits may not always be beneficial, especially for plants growing under sub-optimal environmental conditions. Different lines of evidence have pointed out that changes in organic acid content might greatly improve fruit organoleptic characteristics. It is important to mention, however, that the majority of those advances have been made in model organisms as well as in some plants of agricultural relevance. To successfully transfer these advances to major food crops, which are generally more recalcitrant to genetic manipulation, still remains a great challenge. To further increase our understanding concerning how organic acids affect fruit metabolism we suggest two complementary approaches: (a) it is possible that the usage of introgression Lines (ILs), as the ones developed in tomato (Eshed and Zamir, 1995) could allow us to identify phenotypes with alterations in the levels of TCA cycle intermediates to analyze the relationship between developmental process and primary metabolism; and (b) to genetically engineer fruit-specific inhibitions within TCA cycle enzymes and/or organic acid metabolism/transport to further analyze the metabolic behavior connecting it with fruit development and ripening. Although both approaches have been successfully used already, it seems clear that they also open the opportunity to greatly accelerate the improvement of crops that have clearly lacked the attention they deserve. The adaptation of high-throughput phenotyping alongside more sensitive flux profiling methodologies, is likely to enable us to pursue new avenues of research to increase our understanding of the complex networks governing organic acid function and hormone metabolism in general during fruit ripening.

## Conclusion and Future Perspectives

Although the summary presented here provides a scaffold for understanding the connections between organic acid and hormone metabolism in fruit development, we posit that it is of pivotal importance that these emerging studies should be expanded. More fundamental knowledge is still required to identify further strategies for manipulation that would improve fruit quality and consequently fruit metabolism. It seems reasonable to anticipate that approaches such as genomics, transcriptome, proteomics and metabolomics coupled with genome editing can present itself as an important data generator that would allow the production of a mechanistic map of fruits in general and their association with phytohormones and fruit developmental changes.

## Author Contributions

All authors listed have made a substantial, direct and intellectual contribution to the work, and approved it for publication.

## Conflict of Interest Statement

The authors declare that the research was conducted in the absence of any commercial or financial relationships that could be construed as a potential conflict of interest.
